# The role of growth hormone and IGF-1 in retinopathy: a prospective study of retinopathy in patients with acromegaly and impaired fasting glucose

**DOI:** 10.1186/s13098-022-00806-z

**Published:** 2022-03-05

**Authors:** Tzu-En Wu, Harn-Shen Chen

**Affiliations:** 1 Division of Endocrinology and Metabolism, Department of Medicine, Taipei Veterans General Hospital, No. 201, Sec 2, Shih-Pai Rd, Taipei, Taiwan; 2 School of Medicine, National Yang Ming Chiao Tung University, Taipei, Taiwan; 3 Department of Ophthalmology, Shin-Kong Wu Ho-Su Memorial Hospital, Taipei, Taiwan

**Keywords:** Acromegaly, Growth hormone (GH), Insulin-like growth factor-1 (IGF-1), Impaired fasting glucose, Proliferative retinopathy

## Abstract

**Aims:**

To investigate the effects of the growth hormone (GH)/insulin-like growth factor-1 (IGF-1) axis on the incidence and progression of retinopathy.

**Methods:**

We enrolled 91 patients with acromegaly and 123 subjects with impaired fasting glucose (IFG) between 2008 and 2016 to examine the incidence and prevalence of retinopathy. Patients attended follow-ups in our clinics and underwent examinations according to the national guidelines for diabetes management. Both cohorts attended follow-ups until June 2019.

**Results:**

Both groups had similar HbA1c, cholesterol, and blood pressure levels. However, patients with acromegaly had higher GH (8.05 ± 16.18 vs. 0.78 ± 1.25 ng/mL) and IGF-1 (547.0 ± 342.1 vs. 146.7 ± 51.4 ng/mL) levels than in subjects with IFG. During the follow-up period, 8 patients (8.8%) with acromegaly and 12 patients (9.8%) with IFG developed some degree of retinopathy. Three patients with acromegaly and two with IFG progressed to proliferative retinopathy. Patients with acromegaly had the same incidence of non-proliferative retinopathy (odds ratio [OR] 0.830; 95% CI 0.318–2.164) and a non-statistically significantly higher incidence of proliferative retinopathy (OR 2.461; 95% CI 0.404–14.988).

**Conclusion:**

The data reveals that GH and IGF-1 might play a crucial role in the development of proliferative retinopathy and influence its progression. Therefore, we suggest screening patients with acromegaly should be similar to diabetes patients.

**Supplementary Information:**

The online version contains supplementary material available at 10.1186/s13098-022-00806-z.

## Introduction

Acromegaly is a rare chronic disorder characterized by excessive growth hormone (GH) and insulin-like growth factor 1 (IGF-1) secretion. More than 95% of acromegaly cases are caused by pituitary adenomas [1]. The main effects of GH are mediated by stimulating the production of IGFs [2]. IGF-1 modulates the function of retinal endothelial precursor cells, drives retinal angiogenesis in response to hypoxia, and may play a role in the pathogenesis of proliferative diabetic retinopathy [3]. In addition, there is a large amount of data indicating that an increase in IGF-1 activity may contribute to retinal neovascularization, which is a characteristic sign of proliferative diabetic retinopathy [4, 5].

Neovascularization is the final common pathway of diabetic retinopathy, and IGF-1 has been associated with retinal neovascularization [6]. There have been reports of spontaneous regression of proliferative diabetic retinopathy in women who develop acute total hypopituitarism. Thus, pituitary ablation may be a treatment for vision-threatening retinopathy [7, 8]. Even though IGF-1 may play a pathogenic role in diabetic retinopathy, most research has failed to provide any convincing data regarding the relationship between IGF-1 and the development of retinopathy [9–11].

We have previously reported that serum IGF-1 levels are not associated with the incidence of mild-to-moderate or severe retinopathy in patients with type 2 diabetes [12]. However, in patients with adequate blood glucose control, elevated serum IGF-1 levels are associated with higher cumulative incidence of severe retinopathy. Therefore, we propose that glycated hemoglobin (HbA1c) is a main risk factor for retinopathy and serum IGF-1 influences the development of severe diabetic retinopathy. However, this may be masked by poor blood glucose control [12]. In a previous study, we demonstrated a higher incidence of proliferative retinopathy without non-proliferative retinopathy in patients with acromegaly [13].

We hypothesize that IGF-1 can accelerate the progression of retinopathy from non-proliferative to proliferative and shorten the duration of non-proliferative retinopathy. To examine the influence of the GH–IGF-1 axis on retinopathy, we selected patients with impaired fasting glucose (IFG) to serve as the control group. In addition, we investigated the progression of retinopathy from non-proliferative to proliferative and attempted to clarify the nature of the progression in patients with acromegaly.

## Methods

### Study participants

In this prospective, observational study, we recruited 91 patients with acromegaly who were being regularly monitored at our institution (Taipei Veterans General Hospital) between 2008 and 2016. The diagnosis of acromegaly was made based on clinical findings and confirmed with elevated GH and IGF-1 levels. In addition, we enrolled 123 subjects with IFG diagnoses between 2011 and 2014 to serve as the control group. Patients with IFG underwent an oral glucose tolerance test (OGTT) and the development of diabetes and diabetic complications was monitored at follow-ups. Both cohorts attended follow-ups until June 2019. The Institutional Review Board of Taipei Veterans General Hospital approved the study protocols in 2008 and 2011. Written informed consent was obtained from all participants. This study complies with the Strengthening the Reporting of Observational Studies in Epidemiology (STROBE) statement [14] and the broader EQUATOR guidelines [15].

### Study protocol

To assess glucose homeostasis and disease activity, all patients with IFG and a majority of patients with acromegaly (61/91) underwent an OGTT after an overnight fast. After oral administration of 75 g of glucose, blood was drawn at 0, 30, 60, 90, and 120 min. Blood samples from all time points were assayed for insulin and glucose. Additionally, blood samples from patients with acromegaly were also analyzed for GH. IGF-1, GH, and HbA1c levels at baseline were measured in fasting blood samples. Blood pressure was measured on the same morning the OGTT was performed. All patients had their blood pressure measured from the right arm using an electronic sphygmomanometer while sitting in a relaxed position.

### Funduscopic examinations

We took yearly retinal color photographs of all participants. Photographs were taken with a fundus camera to obtain a central view of the macula and disc at a 45° angle. The photographs were evaluated by experienced endocrinologists blinded to the patients’ medical condition. Our grading system was followed the International Council of Ophthalmology Guidelines for Diabetic Eye Care published in Feb 2014 [16]. We classified the severity of the retinopathy into the following four categories: no apparent retinopathy, mild-to-moderate non-proliferative retinopathy, severe non-proliferative retinopathy, and proliferative retinopathy. Patients with abnormal findings were referred to ophthalmologists for further evaluation. These patients underwent further comprehensive retinal examination, fluorescein angiography, and optical coherence tomography if indicated.

### Laboratory analyses

Serum GH was measured using a sensitive immuno-fluorometric assay (Wallac, Turku, Finland) specific to the 22 kDa GH protein calibrated to the WHO International Reference Preparation 80/505 (detection limit: 0.05 μg/L; inter-assay coefficient of variation [CV]: 1.6%–8.4% between 0.5 and 40 μg/L). Serum IGF-1 was determined using an immuno-radiometric assay (Diagnostic System Laboratory, Webster, TX, USA) with inter- and intra-assay CVs less than 7.4% and 7.0%, respectively. HbA1c was measured using high-performance liquid chromatography (HLC-723G7, Tosoh, Japan) with a reference range of 4.2–5.8%.

### Statistical analyses

The data were analyzed using version 26.0 of IBM SPSS for Windows (SPSS Inc., Chicago, Illinois, USA). All data are expressed as means and standard deviations or as frequencies (percent). The unpaired Student’s *t*‐test was used to compare continuous parameter data between groups, and the Mann–Whitney U test was used to compare non-parametric data between groups. When appropriate, Carter’s test, Yates’ correction, or Fisher’s exact test were used to compare categorical data between groups. Pearson’s χ^2^ test was used to compare nominal and ordinal data from both groups and is reported as a percentage. Kaplan–Meier estimates were assessed to determine the cumulative probability of retinopathy over time. Cox proportional hazards regression models were used to compare the incidence of retinopathy between the groups.

## Results

### Baseline characteristics

A total of 91 patients (42 men, 49 women) with acromegaly were recruited for this study and the mean age at baseline was 49.9 ± 16.0 years. The control group comprised 123 subjects with IFG (33 men, 90 women) and the mean age at baseline was 54.9 ± 9.7 years. The baseline clinical and biochemical data of patients with acromegaly and IFG are shown in Table 1. Notably, patients with acromegaly were younger, predominantly men, taller, heavier, with a higher BMI and higher GH and IGF-1 levels. However, HbA1c, fasting blood glucose, cholesterol, and blood pressure levels were identical in both groups.

**Table 1:** Demographic characteristics of patients with acromegaly and impaired fasting blood glucose

	Acromegaly (n = 91)	Patients with impaired fasting blood glucose (n = 123)	*p* value
Age (years)	49.9 ± 16.0	59.4 ± 9.7	< 0.001
Sex (male/female)	42/49	33/90	< 0.001^#^
Body height (cm)	167.8 ± 12.1	158.6 ± 7.5	< 0.001
Body weight (kg)	74.0 ± 17.2	62.3 ± 10.7	< 0.001
BMI (kg/m^2^)	26.20 ± 4.62	24.75 ± 3.71	0.013
Systolic BP (mmHg)	128.3 ± 16.5	128.3 ± 14.4	0.989
Diastolic BP (mmHg)	77.6 ± 11.8	76.6 ± 9.2	0.471
Fasting GH (ng/mL)	8.05 ± 16.18	0.78 ± 1.25	< 0.001
Fasting IGF-1 (ng/mL)	547.0 ± 342.1	146.7 ± 51.4	< 0.001
Fasting PG (mg/dL)	103.6 ± 20.0	107.8 ± 10.7	0.408
HbA1c (%)	6.22 ± 1.08	6.12 ± 0.48	0.383
Cholesterol (mg/dL)	186.3 ± 27.2	184.4 ± 21.5	0.640
HDL cholesterol (mg/dL)	56.8 ± 26.1	53.2 ± 20.5	0.402
Triglycerides (mg/dL)	111 (86 − 128.5)	109 (79.5 − 171)	0.299*
Creatinine (mg/dL)	0.78 ± 0.41	0.70 ± 0.20	0.087
eGFR (mL/min/1.73 m^2^)	105.7 ± 32.4	103.0 ± 28.5	0.583
Urine albumin-to-creatine ratio (mg/gCr)	12.7 (4.4 − 70.1)	6.8 (4.85 − 16.75)	0.230*

*BP* blood pressure, *HbA1c* glycated hemoglobin, *PG* plasma glucose, *GH* growth hormone.

^#^Fisher’s exact test

*Mann–Whitney U test

**Fig. 1:**
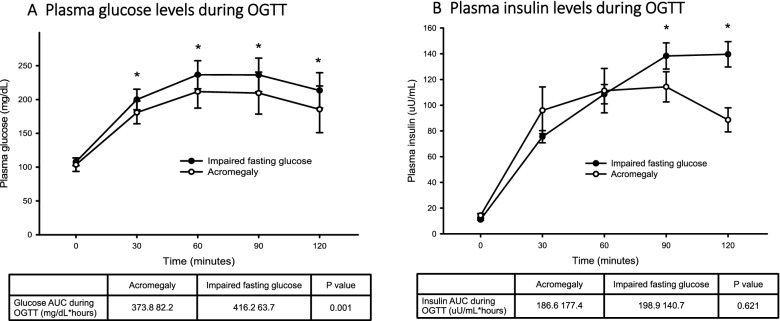
Plasma glucose (**A**) and insulin (**B**) concentrations during the oral glucose tolerance test (OGTT) in patients with acromegaly and subjects with impaired fasting glucose (IFG) (*p < 0.05 between groups)

The plasma glucose and insulin levels during OGTT were presented in Fig. 1. The subjects with IFG had higher glucose levels after oral glucose loading and higher glucose concentration during OGTT, presented with glucose area under curve (AUC) during OGTT (416.2 ± 63.7 vs. 373.8 ± 82.2 mg/dL, p = 0.001). The subjects with IFG had higher insulin levels at 90 and 120 min after glucose loading, however the insulin AUC during OGTT were the same between the two groups (198.9 ± 140.7 vs. 186.6 ± 177.4 uU/mL, p = 0.621).

### The cumulative probability of retinopathy

**Fig. 2:**
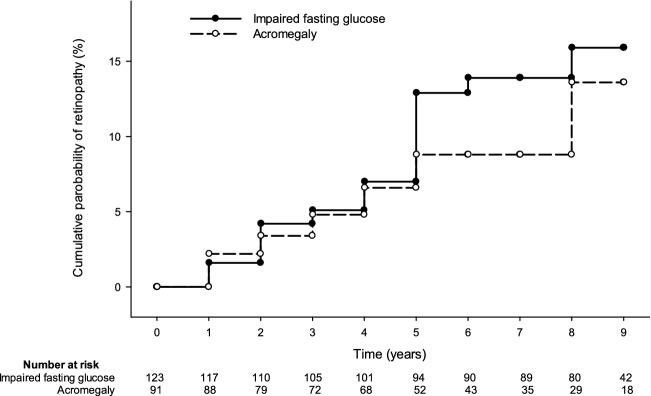
Cumulative probability of retinopathy in patients with acromegaly and subjects with IFG during the 8 year follow-up period

The median follow-up period was 64 months (interquartile range [IQR] 39–109 months) for patients with acromegaly and 85 months (IQR 72–94 months) for patients with IFG. During the follow-up period, 8 patients (8.8%) with acromegaly and 12 (9.8%) with IFG developed some degree of retinopathy (Fig. 2). Table 2 shows the incidence of any retinopathy, non-proliferative retinopathy, and proliferative retinopathy in patients with acromegaly and IFG. Three patients (3.3%) with acromegaly and two (1.6%) with IFG progressed to proliferative retinopathy. One patient in each group was diagnosed with proliferative retinopathy without diagnosing as no non-proliferative retinopathy during the study period, however they also had other signs of non-proliferative diabetic retinopathy. Although patients with acromegaly had the same incidence of non-proliferative retinopathy (odds ratio [OR] 0.963; 95% CI 0.373–2.485) and non-statistically significantly higher incidence of proliferative retinopathy (OR 2.461; 95% CI 0.404–14.988).

**Table 2:** Incidence of retinopathy in patients with acromegaly (n = 91) and impaired fasting glucose (n = 123)

	Acromegaly	Impaired fasting glucose	Odds ratio	95% confidence interval
	n (%)	n (%)		
No apparent retinopathy	84 (92.3)	107 (87.0)		
Any level of retinopathy	8 (8.8)	12 (9.8)	0.963	0.373–2.485
Non-proliferative retinopathy	7 (7.7)	11 (8.9)	0.830	0.318–2.164
Proliferative retinopathy	3 (3.3)	2 (1.6)	2.461	0.404–14.988

One patient in each group was diagnosed with proliferative retinopathy without diagnosing as no non-proliferative retinopathy during the study period, however they also had other signs of non-proliferative diabetic retinopathy

### Baseline clinical characteristics of patients with proliferative retinopathy

The baseline clinical and biochemical characteristics of patients who developed proliferative retinopathy during the study period are shown in Table 3. Additional file 1: Figure S1 showed the eye fundi pictures of the 5 patients with proliferative retinopathy. Patients with acromegaly with proliferative retinopathy had higher GH, IGF-1, and HbA1c levels than those without proliferative retinopathy. In contrast, patients with IFG who had proliferative retinopathy had similar GH and IGF-1 levels and traditional risk factors for retinopathy compared to those without proliferative retinopathy.

**Table 3:** Baseline clinical characteristics of patients with proliferative retinopathy

	Age (years)	Sex	Fasting GH (ng/mL)	Fasting IGF-1 (ng/mL)	HbA1c (%)	Systolic BP (mmHg)	Diastolic BP (mmHg)	Cholesterol (mg/dL)	Triglycerides (mg/dL)	UACR (mg/gCr)
Acromegaly-04	34	Female	17.9	1840	7.9	124	77	190	96	92.6
Acromegaly-52	55	Female	15.4	1055	6.9	114	69	163	101	15.8
Acromegaly-54	58	Female	34.2	906	7.1	120	61	172	62	12.2
Impaired fasting glucose-307	61	Female	0.3	123	6.6	154	82	78	72	16.7
Impaired fasting glucose-428	72	Male	0.1	138	6.2	112	67	228	312	5.2

*HbA1C* glycated hemoglobin, *GH* growth hormone, *BP* blood pressure, *UACR* urine albumin-to-creatine ratio (mg/gCr)

## Discussion

We followed 91 patients with acromegaly and 123 patients with IFG for more than 8 years to study and compare the incidence of retinopathy in patients with abnormal glucose homeostasis, who are thus at risk for developing retinopathy. To the best of our knowledge, this is the first study to compare the incidence of retinopathy in these patients. Both groups had similar HbA1c, cholesterol, and blood pressure levels. A significant intergroup difference was higher GH (8.05 ± 16.18 vs. 0.78 ± 1.25 ng/mL) and IGF-1 (547.0 ± 342.1 vs. 146.7 ± 51.4 ng/mL) levels in patients with acromegaly. During the follow-up period, 8 patients with acromegaly (8.8%) and 12 with IFG (9.8%) developed some degree of retinopathy. Three patients with acromegaly (3.3%) and two with IFG (1.6%) progressed to proliferative retinopathy. Patients with acromegaly were noted to have the same incidence of non-proliferative retinopathy (OR 0.830; 95% CI 0.318–2.164) and a non-statistically significantly higher incidence of proliferative retinopathy (OR 2.461, 95%; CI 0.404–14.988).

Retinopathy is the most common complication in diabetes patients and can even develop during the prediabetes stage. Patients with acromegaly usually have elevated GH and IGF-1 levels, which can lead to insulin resistance and diabetes [17]. GH has been considered pathogenic for diabetes for nearly a century, and excessive GH secretion can cause microvascular complications, like retinopathy [18]. Füchtbauer et al. [19] conducted a study to evaluate the morphology of retinal vessels in patients with acromegaly and the prevalence of diabetic retinopathy in patients with acromegaly and diabetes. They reported a higher number of retinal blood vessel branches in patients with acromegaly. However, there was not a high prevalence of diabetic retinopathy in patients with acromegaly and diabetes [19].

Risk factors for diabetic retinopathy include the time since onset of diabetes, high blood glucose, high blood pressure, dyslipidemia, and insulin resistance [20, 21]. Yau et al. [22] conducted a systematic study to examine the overall prevalence and risk factors for diabetic retinopathy in patients with diabetes. The overall prevalence of any retinopathy was 34.6%, and the prevalence of proliferative retinopathy was 6.96%. The prevalence increase was associated with the time since onset of diabetes and HbA1c and blood pressure levels [22]. Song et al. [23] conducted a systematic review and meta-analysis to investigate the prevalence and risk factors for retinopathy in China. The observed prevalence for retinopathy was 1.14% in the general population and 0.07% for proliferative retinopathy. In patients with diabetes, the combined prevalence for any retinopathy was 18.45%, and the combined prevalence for proliferative retinopathy was 0.99% [23]. We retrospectively analyzed 43 patients with acromegaly and 129 age- and gender-matched type 2 diabetes patients [13]. We found that 9.3% of patients with acromegaly (4/43) and 9.3% of patients with type 2 diabetes (12/129) had proliferative retinopathy. However, non-proliferative retinopathy was not observed in patients with acromegaly, while it was observed in 25.9% of patients with type 2 diabetes (33/129) [13]. Even though non-proliferative retinopathy is not observed in patients with acromegaly, the frequency of proliferative retinopathy is the same as in patients with type 2 diabetes [13]. Füchtbauer et al. [19] conducted a cross-sectional study to assess the prevalence of retinopathy in 39 patients with long-term acromegaly and diabetes in Sweden. They diagnosed five cases (13%) of retinopathy (two cases of proliferative diabetic retinopathy and three cases of proliferative diabetic retinopathy) and concluded that the prevalence of retinopathy is not higher in patients with acromegaly compared to patients with diabetes [19]. In the current 8 year follow-up study, retinopathy was diagnosed in 8 patients (8.8%) with acromegaly and 12 patients (9.8%) with IFG. According to our research and previous reports, we may assume that the prevalence of non-proliferative retinopathy in patients with acromegaly is higher than in the general population, lower than in patients with diabetes, and the same as in patients with IFG [13, 20–23]. Additionally, the prevalence of proliferative retinopathy in patients with acromegaly is higher compared to the general population and patients with prediabetes and may be similar to the prevalence in patients with diabetes [13, 20–23].

This study’s strengths include the recruitment of a relatively high number of patients with acromegaly and the regular follow-ups. This study is the first to compare the prevalence of retinopathy in patients with acromegaly and IFG. Both groups had identical major risk factors for retinopathy as reflected by their HbA1c, cholesterol, and blood pressure levels. The only significant intergroup difference was their GH and IGF-1 levels, enabling us to precisely investigate their effects on retinopathy development. Nevertheless, our study was limited by presenting only a small event rate for proliferative retinopathy, which prevents confirmation of our hypothesis. Therefore, including more participants and a longer follow-up period is required to confirm our hypothesis.

In conclusion, although not statistically significant, patients with acromegaly appear to have the same incidence of non-proliferative retinopathy and a non-statistically significantly higher incidence of proliferative retinopathy compared to patients with IFG. Our data revealed that GH or IGF-1 may play a crucial role in the development of proliferative retinopathy and may influence the progression of retinopathy. Although a future study with more patients and a longer follow-up period is warranted, the data suggest that patients with acromegaly, like patients with diabetes, should be screened for retinopathy.

## Supplementary Information

**Additional file 1: Figure S1.** shows the eye fundi pictures of the 5 patients with proliferative retinopathy.

## Data Availability

Additional file 1 with the file name of Acromegaly (6) and IFG has GH data 2021-04.pdf.
