# Supplementary material for: The role of growth hormone and IGF-1 in retinopathy: a prospective study of retinopathy in patients with acromegaly and impaired fasting glucose
Source: Diabetol Metab Syndr. 2022 Mar 5;14:38. doi: 10.1186/s13098-022-00806-z (PMC8898474; doi:10.1186/s13098-022-00806-z)
Supplement: Supplementary file 1 — Additional file 1: Figure S1. shows the eye fundi pictures of the 5 patients with proliferative retinopathy. [file 13098_2022_806_MOESM1_ESM.pptx]

## Slide 1
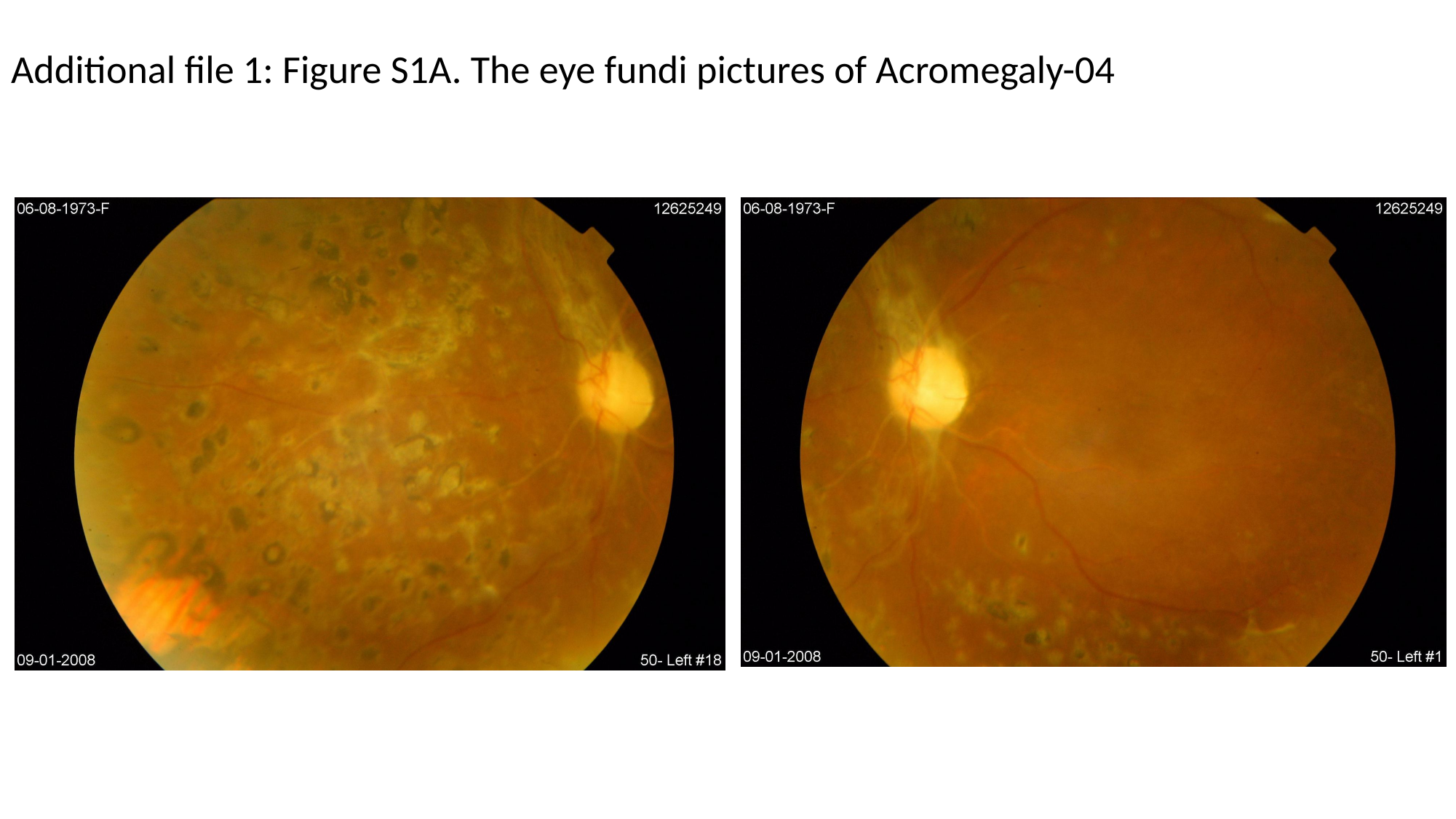

# Additional file 1: Figure S1A. The eye fundi pictures of Acromegaly-04

## Slide 2
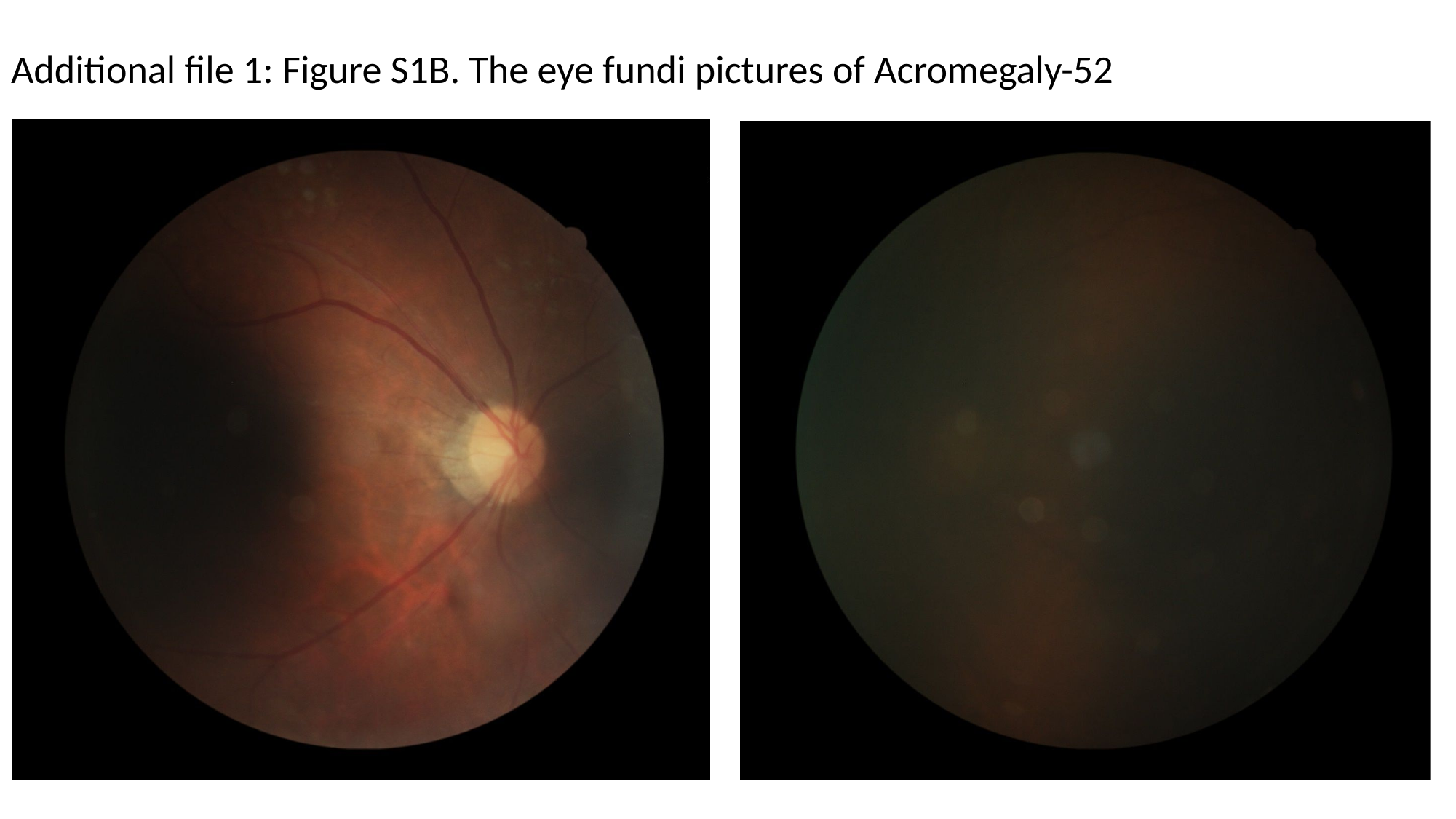

# Additional file 1: Figure S1B. The eye fundi pictures of Acromegaly-52

## Slide 3
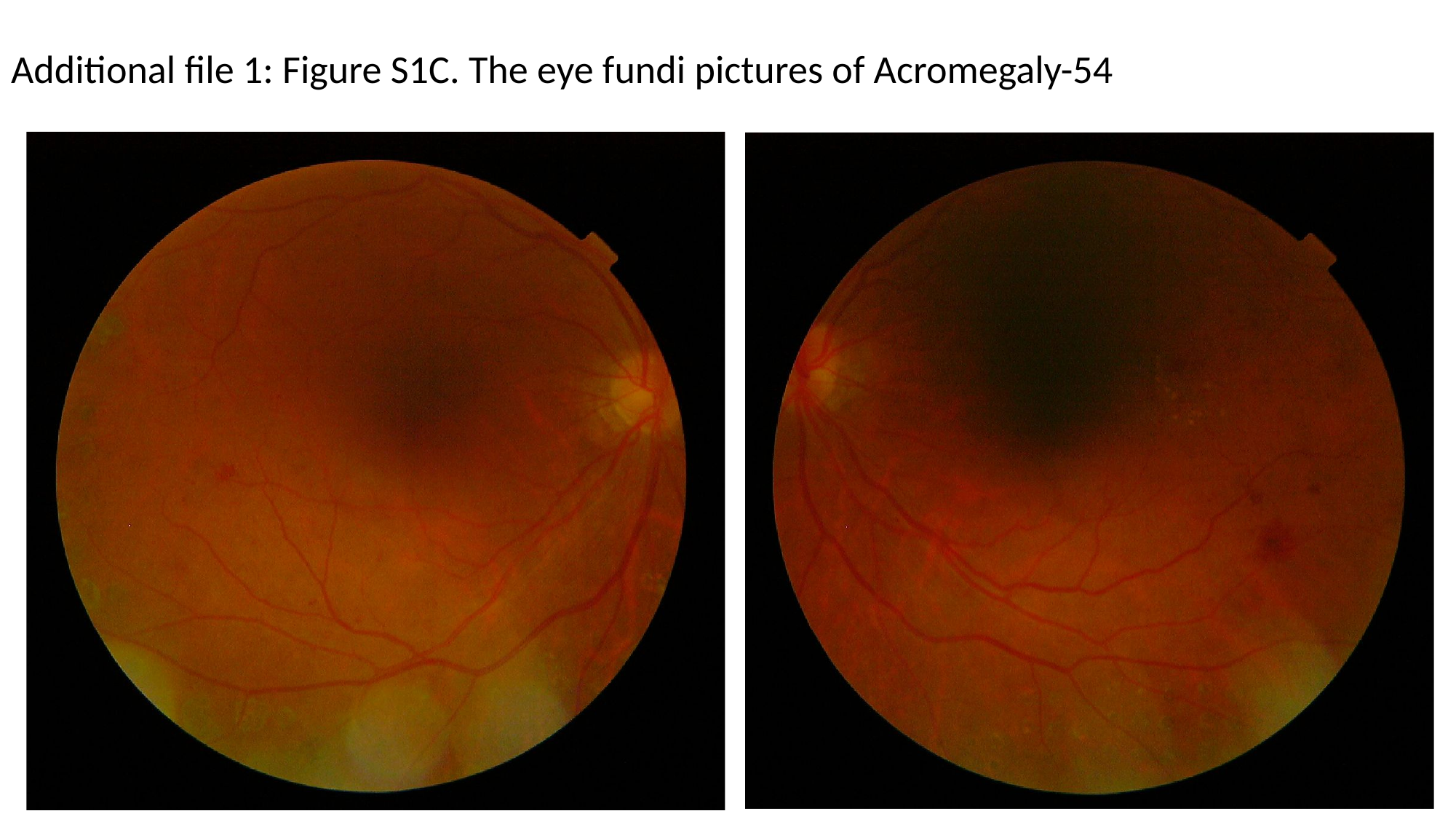

# Additional file 1: Figure S1C. The eye fundi pictures of Acromegaly-54

## Slide 4
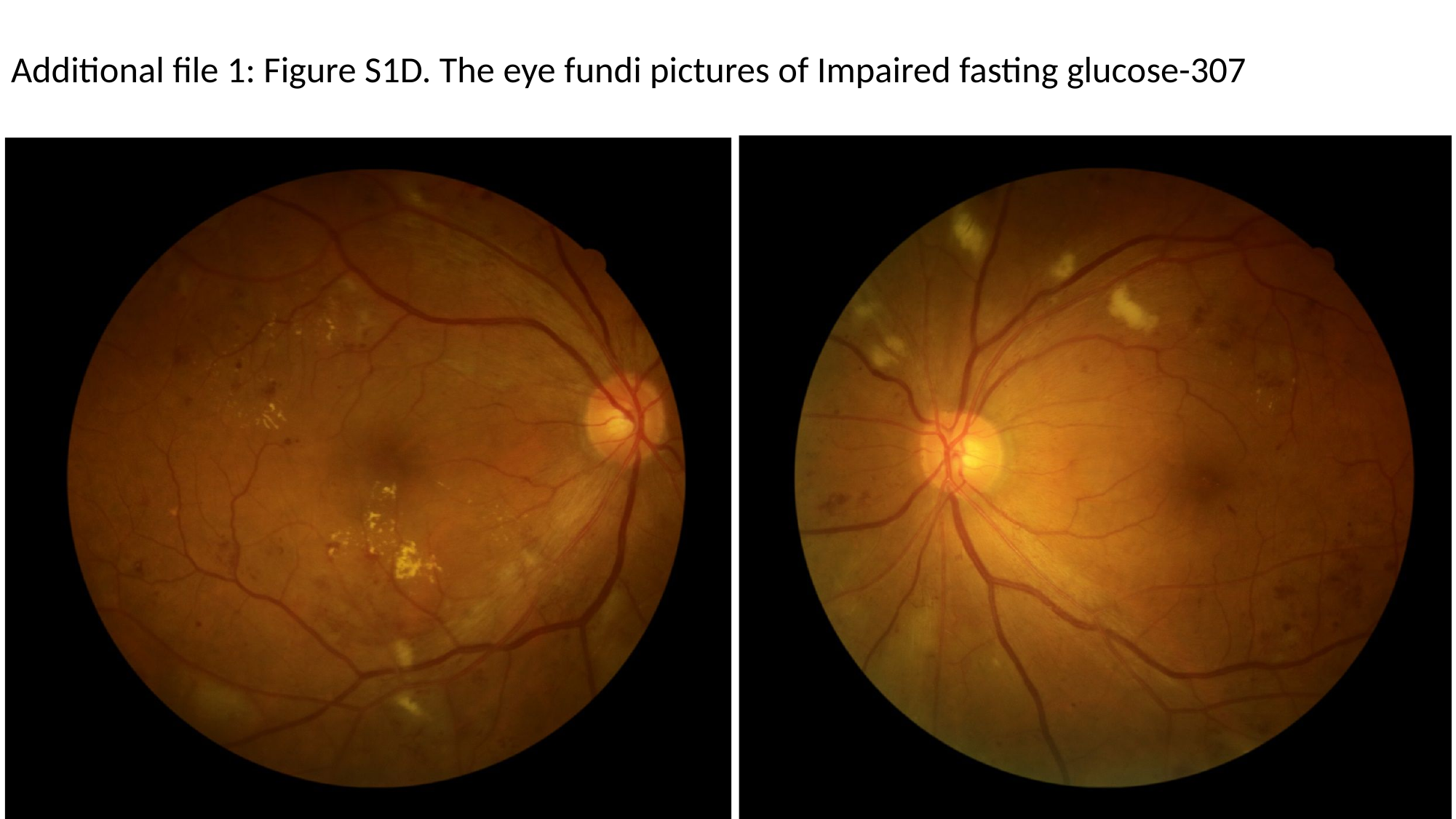

# Additional file 1: Figure S1D. The eye fundi pictures of Impaired fasting glucose-307

## Slide 5
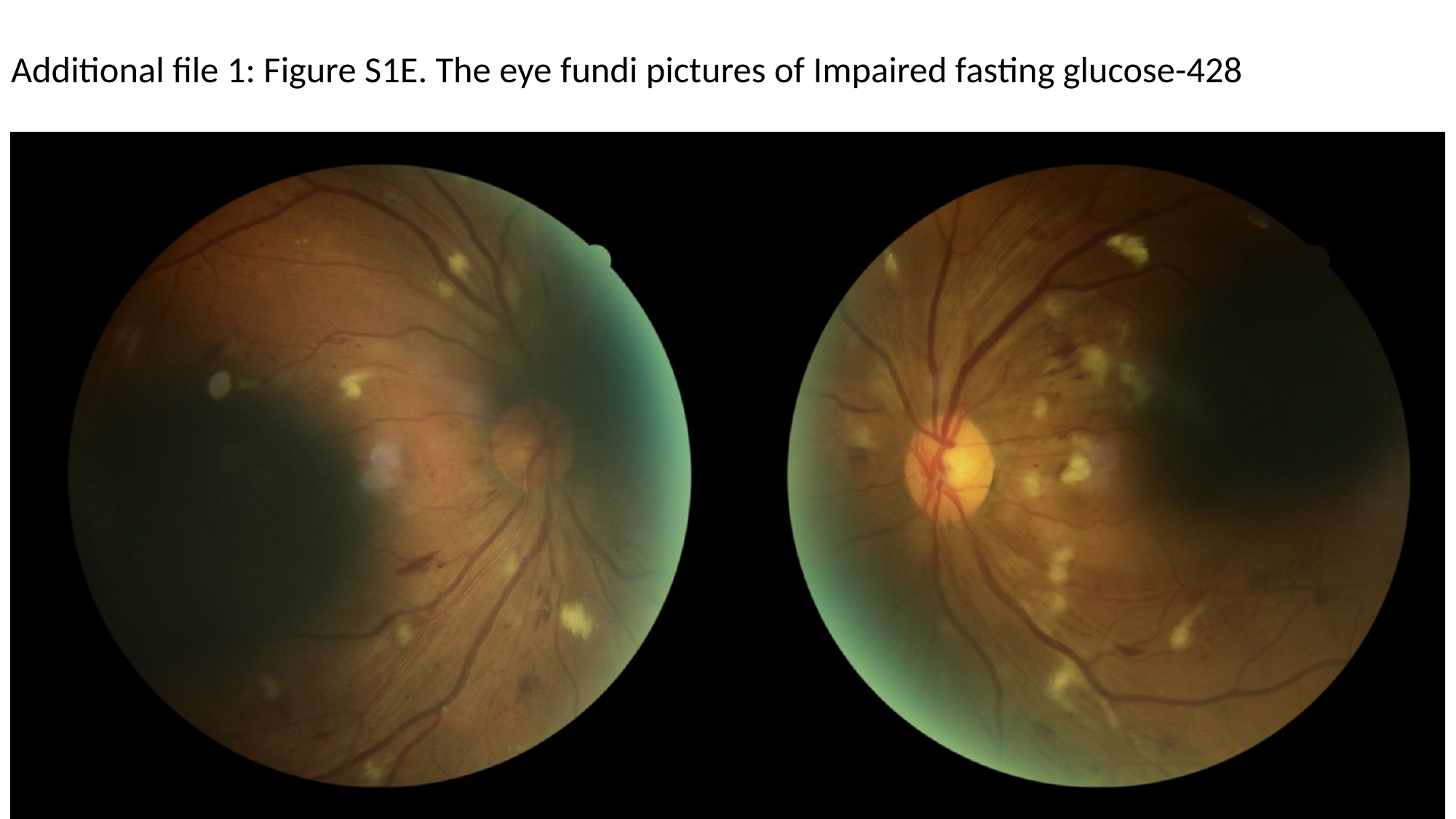

# Additional file 1: Figure S1E. The eye fundi pictures of Impaired fasting glucose-428
